# Green Pit Viper Envenomations in Bangkok: A Comparison of Follow-Up Compliance and Clinical Outcomes in Older and Younger Adults

**DOI:** 10.3390/toxins14120869

**Published:** 2022-12-10

**Authors:** Rittirak Othong, Thanaphat Eurcherdkul, Prasit Chantawatsharakorn

**Affiliations:** 1 Department of Emergency Medicine, Faculty of Medicine Vajira Hospital, Navamindradhiraj University, Bangkok 10300, Thailand; 2 Department of Emergency and Forensic Medicine, Taksin Hospital, Bangkok 10600, Thailand

**Keywords:** *Trimeresurus albolabris*, *Trimeresurus macrops*, elderly, senior, aging, morbidity, mortality, snakebite, coagulopathy, thrombocytopenia

## Abstract

We compared older and younger adults envenomated by the green pit viper (GPV) with regard to the following: follow-up compliance, elapsed time between envenomation and emergency department (ED) visit, and clinical/treatment outcomes. This was a two-site retrospective cohort study. We searched hospital electronic medical databases between January 2011 and December 2021. Patients aged 15 and above were eligible if they had a history of snakebite and had at least two VCT and/or platelet count results in their medical records. After the search, 1550 medical records were reviewed and 760 cases were found to be eligible for analysis. In total, 205 cases (27.0%) were ≥60 years old. The median ages in the younger and older groups were 40 (26–51) and 68 (64–75) years, respectively. The median elapsed times from bite to the ED were 47 (30–118) vs. 69 (35–150) min (*p*-value = 0.001). Overall, 91.3% of all cases were managed as out-patient cases and were eligible for follow-up appointments. The rate of out-patient follow-up at 72 ± 12 h in the older patients was significantly higher (43.2%) than in the younger adult patients (32.4%) (*p*-value = 0.01). Regarding the clinical/treatment outcomes, the rates of coagulopathy, antivenom administration, and hospital admission were not statistically different between both groups.

## 1. Introduction

Snakebites are still an issue in Thailand’s public health system. In 2018, the Department of Disease Control released a report that an average of 5035 people were bitten by venomous snakes per year between 2009 and 2018. When the snake could be identified, for the year 2018, the green pit viper was found to be the most common at 31.9% [1]. In Bangkok, the most common venomous snakebites (>90%) were from the green pit viper (GPV) [2]. There are only two GPV species in the Bangkok area, *Trimeresurus albolabris* (the white-lipped pit viper) and *T macrops* (the big-eyed pit viper) [3,4]. One major component of the GPV’s venom resembles thrombin, a so-called thrombin-like enzyme (TLE), GPV-TL1/albolabrase [5,6,7]. To form a firm clot, thrombin cleaves both fibrinopeptide A and B from fibrinogen, but the TLE in the venom only cleaves fibrinopeptide A and not B, producing abnormal fibrin polymerization and a friable fibrin clot [8]. In addition, this process consumes fibrinogen, causing low fibrinogen levels in the blood, and this can contribute to aberrant systemic bleeding, such as bleeding from the oral mucosa and from the gastrointestinal or urinary tracts [9]. Other hematologic abnormalities caused by GPV envenomation are thrombocytopenia, which is due to C-type lectins and alboaggregin B and D, which promote platelet aggregation, and also disintegrin, which potentiates platelet dysfunction. Local tissue reactions are another concern following a bite, which can be the result of an interplay of multiple enzymes in the venom, such as metalloproteinases, phospholipases A2, and collagenases [10,11].

Regarding treatment, after a GPV bite patient arrives in the ED, blood should be taken for a venous clotting time (VCT) or 20 WBCT (20 min whole blood clotting time), and a complete blood count, including platelet count, at the least. The following criteria are used for antivenom administration and admission: (1) systemic bleeding; (2) VCT lasting more than 20 min, or unclotted blood at 20 min (20WBCT), or INR ≥ 1.2; (3) platelets < 100,000 /mm^3^; (4) severe local swelling or impending compartment syndrome; (5) compartment syndrome [12,13,14]. If none of the above criteria are met, the patient will be scheduled for out-patient follow-up on a daily basis for 3 days after the bite.

As of 2015, Thailand has an aging society, with seniors over 60 accounting for 15% of the overall population. It is expected that by 2035 Thailand will have become even more of an aged society, with more than 30% of the population over 60 [15]. Older people undergo physiological changes and may have underlying medical disorders or use medications, such as anticoagulants and antiplatelets, that put them at increased risk of bleeding [16,17].

In a survey study that collected data from national surveys of older people from three countries in Southeast Asia, Myanmar, Vietnam, and Thailand, it was revealed that percentages of solo living in older adults have been rising. From this study, compared with two decades ago, solo living among older people in Thailand was found to have increased more dramatically compared with Myanmar and Vietnam. It was found that 16% of older Thai people had no children and that, for those who had children, 40% of their children lived somewhere else and not close to them. Being alone can restrict personal assistance when it is in need [18]. It is unclear whether advanced age can affect patients’ abilities to seek medical care after a snakebite or affect clinical/treatment outcomes.

Very few studies exist at present regarding snakebites in this vulnerable population and even fewer have compared follow-up compliance and clinical/treatment outcomes among younger adults and older ones. In this study, we aimed to compare follow-up compliance, elapsed time from bite to emergency department visit, clinical symptoms, and clinical/treatment outcomes between the younger and older adults in our study who had been bitten by green pit vipers.

## 2. Results

### 2.1. Patient Characteristics 

**Figure 1:**
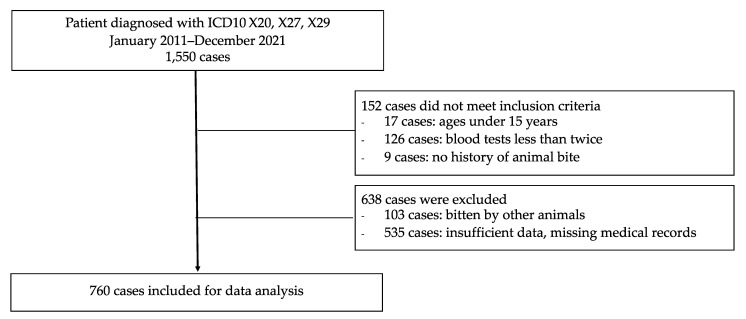
Number of patients included in the study.

**Figure 2:**
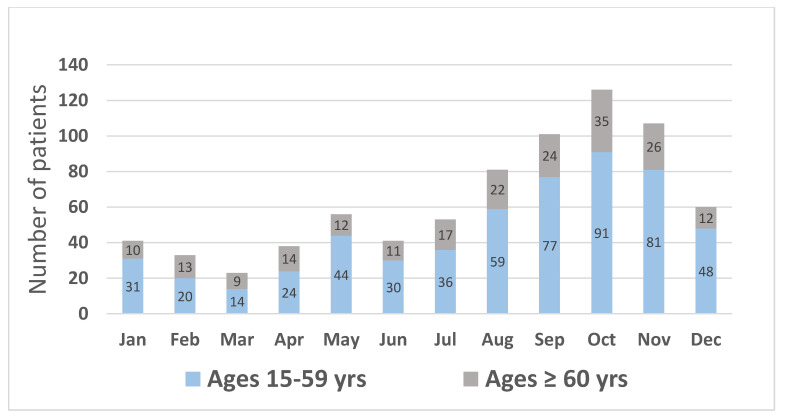
Number of patients by month and proportions of younger and older patients from January 2011 to December 2021.

Between January 2011 and December 2021, 1550 cases were diagnosed with ICD10 X20, X27, or X29. From a total of 1550 cases, 790 cases were excluded due to not meeting the inclusion criteria or meeting at least one of the exclusion criteria (Figure 1). Finally, 760 cases were included for data analysis. The prevalence of bites per month was high during August and November in both age groups (Figure 2). Among the 760 cases, 205 were 60 years old or older (27.0%). In these older patients, more than half had at least one underlying disease, hypertension being the most common (Table 1). Medical histories of hypertension in this group were consistent with physical examinations demonstrating higher systolic blood pressure as compared with the systolic blood pressure results for the younger group.

### 2.2. Bite Information (Table 1)

Overall, the frequency of undetermined bites was low at 5.1%. The remaining 94.9% of bites were further classified as definite GPV bites (43.0%), probable GPV bites (22.6%), or possible GPV bites (29.3%) (definitions are provided in the Materials and Methods section). The median elapsed time from bite to ED arrival was significantly shorter, by 22 min, among the younger patients as compared with the older patients. The majority in both groups were bitten outdoors around the house or away from the house, even in the older adults. Younger adults were bitten more often in the feet (67.2%), whereas the most common site of bites in older adults was the hands (45.9%), and the difference was significant (*p*-value < 0.001). 

### 2.3. Clinical Features (Table 1)

The degrees of local reactions, including hemorrhagic blebs and wound necrosis, between the two groups were similar. Systemic signs and symptoms were also not different. Abnormal or systemic bleeding was not found at all in either group, even though more of the older patients had taken antiplatelets, anticoagulants, or both (7.6 folds, *p*-value < 0.001). A younger patient had anaphylaxis (rash, dyspnea, nausea/vomiting) from envenomation upon arrival at the ED. No one had wheezing.

### 2.4. Laboratory Results (Table 2 and Supplementary Table S1)

For blood tests, the first test was performed immediately after ED arrival or about 2 h after the bite; the second was performed around 2–3 h after the first test. If all the results came back as normal, the patient was discharged and blood would be tested again at 24±12 h after the bite (the third test), a fourth time at 48 ± 12 h, and a fifth time at 72 ± 12 h after the bite. Blood test results were not significantly different between younger and older patients. 

**Table 2:** Comparison of clinical outcomes and treatment outcomes in the younger and older adult patients.

	Total (n= 760)	Ages < 60 Years (n = 555)	Ages ≥ 60 Years (n = 205)	*p*-Value
**Clinical Outcome**				
Abnormal laboratory results (at any time point and by any test)	83/760 (10.9)	56/555 (10.1)	27/205 (13.2)	0.23
Abnormal VCT or 20WBCT	54/760 (7.1)	37/555 (6.7)	17/205 (8.3)	0.44
Abnormal platelet (<100,000)	16/707 (3.3)	10/514 (2.0)	6/193 (3.1)	0.35
Abnormal fibrinogen (<100)	5/78 (6.4)	4/51 (7.8)	1/27 (4.7)	0.48
Abnormal INR (>1.2)	19/502 (3.8)	11/349 (3.2)	8/153 (5.2)	0.26
Received antivenom	39 (5.1)	28 (5.1)	11 (5.4)	0.86
**Treatment outcome**				
Disposition after initial ED management				
Observation (details of disposition after ED observation are below)	738 (97.1)	535 (96.4)	203 (99.0)	0.41
Discharge	6 (0.8)	5 (0.9)	1 (0.5)	
Hospital admission	13 (1.7)	12 (2.1)	1 (0.5)	
Referral	2 (0.3)	2 (0.4)	0 (0.0)	
Left against medical advice	1 (0.1)	1 (0.2)	0 (0.0)	
Disposition after ED observation	738 (97.1)	535 (96.4)	203 (99.0)	
Discharge	668 (90.5)	486 (90.8)	182 (89.7)	0.77
Hospital admission	30 (4.1)	20 (3.7)	10 (4.9)	
Referral	40 (5.4)	29 (5.4)	11 (5.4)	
Hospital admission (at any point)	67 (8.8)	50 (9.0)	17 (8.3)	0.76
In-hospital discharge status				
Alive with complete recovery	67 (8.8)	50 (9.0)	17 (8.3)	0.76

### 2.5. Treatment 

Most patients who were bitten by a green pit viper were treated in the ED and then discharged home for out-patient follow-up appointments (91.3%). Only 8.8% of patients with GPV bites were admitted due to risk of systemic bleeding or coagulopathy. A total of 39 cases were administered antivenom. None of the bitten cases in either age group underwent any invasive procedure or any kind of surgical intervention for treatment. None of the patients received vasopressors. No one received blood components because nobody had systemic bleeding. There were no deaths in this study.

### 2.6. Follow-Ups

Overall, the follow-up compliance at 72 h was poor at about one third. However, it was significantly higher in the older compared with the younger patients, either including (43.2% vs. 32.4%, *p*-value = 0.01) or excluding (43.4% vs. 33.1%, *p*-value = 0.02) undetermined bite cases (Table 3). Younger adults were lost to follow-up mostly on day 2, but in the older patients the majority were lost to follow-up equally on days 2 or 3 (Table 4). In comparing the two sites of data collection, we found that follow-up compliance was significantly higher at Vajira Hospital (4.5 folds, *p*-value < 0.001), where toxicology rotation and consultation were available on a daily basis (Supplementary Tables S2 and S3). 

## 3. Discussion

In this study, for the two medical centers in Bangkok, the difference in the follow-up compliance at 72 ± 12 h between the two age groups of snake-bitten patients was significant (*p*-value = 0.01). Surprisingly, the older patients had better compliance with follow-up. Older patients might have had more available time for follow-up appointments compared with the younger adults, since the older patients were likely retired from work (in Thailand, retirement age is 60 years old), unlike the younger adults, who may be busy with daily living, such as working for income or taking care of children or spouses. The older patients in this study might still be quite active as well, since most of the bites occurred outdoors around the house or outside away from the house (Table 1, bite scenes). This might imply that the older patients could ambulate well; as a result, with public transportation in Bangkok, it would not be difficult for them to travel to the hospital by themselves. The follow-up rate in older adults might have been lower if the setting was instead in a rural area, where public transportation may not be readily available and where older adults may be more dependent on their relatives to take them to hospital. As pointed out in a previous study, living in an urban area provided a higher quality of life among older adults with hypertension [19]. To confirm our theory for the next project, we plan to incorporate mode of arrival and Charlson comorbidity index into the medical records for those with snakebites to better understand the differences between these two age groups with respect to hospital follow-up appointments. 

Even though the older patients had higher compliance in follow-up compared with the younger ones, we found that the younger patients had a shorter elapsed time between a bite and ED arrival. The reason for this was unclear, because this was a retrospective study and information regarding the reason for this result could not be ascertained from the data we could gather. We postulated that, even though the older patients seemed to be independent, they preferred someone to accompany them to the hospital at first after the bite occurred. Finding a person for such a task would require some time, including the time the person took to come and pick up the patient. Nonetheless, even though these older adults arrived later at the ED compared with the younger patients, adverse outcomes of treatment were not different. The venom of the GPV requires time to consume fibrinogen and usually coagulopathy does not occur within the first hour after a bite [3,9], so the difference in the median times between the two groups of 22 min would not have affected clinical outcomes (Table 1, elapsed time since bite). 

The rate of complete follow-up at Vajira Hospital was significantly higher than the rate of complete follow-up at Taksin Hospital (*p*-value < 0.001). This might be because it was mandatory for all residents to consult a medical toxicologist for envenomation and intoxication review at Vajira Hospital. Medical toxicology consultation as well as residency toxicology rotation became available at Vajira Hospital in October 2012. If a patient was lost to follow-up, the in-service medical toxicologist would ask resident rotators to call the patient to come back to the ED for follow-up. This might be a reason that the follow-up rate at Vajira Hospital was significantly higher than that at Taksin Hospital. This was supported by a study “Assessing the effect of a medical toxicologist in the care of rattlesnake-envenomated patients”; medical toxicologist care can be advantageous for those bitten by venomous snakes [20].

The proportion of hypertension in older adults was 55.6%, which was similar to the figure reported in a previous study on snake bites in older patients which gathered data from the ToxIC (Toxicology Investigators Consortium) data registry [21]. Most of the bites in the older patients occurred around the house, and the hands were the most common site of bites, as discussed above. Due to older patients’ retirement status, it seems that activities in their daily living may have changed to doing light jobs, such as gardening, cleaning, lawnmowing, and decorating the house [22]. These activities can require reaching hands into bushes and trees. Green pit vipers are normally found in trees or wrapped around branches and on the ground [23]. As these snakes are green, they can be difficult to spot in bushes or trees, or on the ground amidst green leaves. Based on our study, this can be seen to indicate a special precaution for older patients, and this information should be passed on to older adults so that they might be made aware and look carefully when working or walking outdoors, especially in the dark or when reaching into bushes and trees, and also use protective equipment, such as gloves. Regarding the severity of local reactions, most cases had a mild or no local reaction (70.4%). After we stratified our cases based on certainty of GPV identification, even in certain GPV bites the majority of patients (66.3%) still had mild local reactions (Supplementary Table S5.1). There are two GPV species in Bangkok, *T albolabris* and *T macrops*. It is well-known that local reactions to *T albolabris* bites are more severe than those to *T macrops* [4]. From the experience of one of our investigators, who has been a medical toxicologist and has seen GPV bite patients at bedside for more than 10 years, most of the GPV specimens brought in with the bite victims were from *T macrops.* This may explain why most of our cases had mild local reactions. Systemic symptoms of GPV bites other than hematologic features, such as rash, dyspnea, chest pain, abdominal pain, and nausea/vomiting, were quite rare (<1%) (Table 1). 

Adverse clinical outcomes, such as abnormal laboratory results, bleeding tendency, or compartment syndrome, were not found in this study (Table 2 and Supplementary Table S2) and were not significantly different between the two groups, even though the older adult patients had more underlying diseases and had taken more antiplatelets/anticoagulants (Table 1). Even though 11% of total cases had at least one hematologic abnormality, only 5% of them received antivenom. This was probably because the abnormality was frequently not severe. For example, the WHO guidelines recommend administering antivenom when the platelet count is below 100,000 /mm^3^ [12]. However, our local recommendation did not indicate antivenom administration until the platelet count is below 50,000 /mm^3^ [14]. This also applied to VCT. Our local guideline used to recommend antivenom administration when VCT > 30 min after GPV bite [24,25]. The 20WBCT was then introduced to our practice according to the WHO guideline that if blood does not clot at 20 min, antivenom should be given. After the 20WBCT gained popularity, the recommendation by local experts for giving antivenom was changed for the VCT to give antivenom when VCT > 20 min instead of 30 min to prevent confusion due to using different time cut-offs between the 20WBCT and the VCT, even though the methods for the two tests were different. In our own practice, when VCT > 20 min but still < 30 min, we ordered another test, such as fibrinogen level, PT/INR, or 20WBCT. Both PT/INR and 20WBCT had high sensitivity (86%) and specificity (96%) when using fibrinogen level < 100 mg/dL as an indicator for severe hypofibrinogenemia from GPV envenomation [26]. If any of these tests were abnormal (fibrinogen < 100 mg/dL, INR ≥ 1.2, abnormal 20WBCT), we usually recommended giving antivenom. 

No invasive procedures or surgical interventions or worsening cases that needed blood components or vasopressor drugs were seen in the admission cases. There was no mortality at all among those admitted to the hospitals, as in other reports [3,10,27].

## 4. Limitations

Since this study was conducted retrospectively, there were some limitations. First, there were insufficient data for a number of medical records; especially in the early years of data collection, registration of ICD10 did not match the patient history, and there were some missing medical records. Some information that could be explanatory for compliance and elapsed time since bite to ED arrival, such as functional status or frailty score for the older patients, living status (alone or with relatives), and mode of arrival, could not be identified. Sixty-five percent of patients did not complete follow-up to day 3 (Table 3), and this may have affected the clinical and treatment outcomes presented in our study. However, for those who were lost to follow-up, they were lost to follow-up since day 2, and only 27% were lost to follow-up at 24 h (Table 4). Coagulopathy and thrombocytopenia, as systemic effects of GPV bites, mostly occurred within 24 h (Supplementary Table S2). As a result, we believe that those who were lost to follow-up were unlikely to have been severely envenomated and less likely to have had severe coagulopathy or systemic bleeding. They also lived not far from our hospitals, as indicated by the median elapsed time from bite to ED arrival, which was 55 min (Table 1). If they had severe coagulopathy/thrombocytopenia to a degree sufficient to cause systemic bleeding, they could reach our hospitals easily and we would not miss those cases with revisits or those with serious outcomes. 

## 5. Conclusions

Older adults took more time to reach the ED after a GPV bite but had a higher rate of completed follow-up at 72 ± 12 h. Even though the older patients had more underlying diseases, they had favorable outcomes that were similar to outcomes in the younger patients. 

## 6. Materials and Methods

This was a two-site retrospective cohort study. We searched the electronic medical databases of two hospitals, one university hospital (900 beds with tertiary care) and one university-affiliated hospital (500 beds with secondary care) in Bangkok using the ICD10 (International Classification of Diseases, 10th edition) codes X20, X27, and X29 between January 2011 and December 2021. Patients aged 15 years and above were eligible for this study if they had a history of snakebite and had at least two VCT and/or platelet count results. We excluded cases with a known type of animal bite that was clearly identified as being not from a GPV, such as a bite from a cobra, a non-venomous snake, or a non-venomous animal. Patients with insufficient data in the medical records or those with missing medical records were also excluded. 

The following data were collected: general information, such as sex, age, geographic location of bite, underlying disease, tetanus vaccination history, use of antiplatelets/anticoagulants, bite site, date and time of bite, date and time of ED arrival, initial vital signs, clinical manifestations, and severity of local reaction. For the investigation, we collected VCT or 20WBCT, platelet count, PT/INR, and fibrinogen level (if available). For treatment and outcomes, the following were collected: ED disposition, antivenom administration, admission status, final hospital discharge status, administration of blood components, unplanned ED revisit, surgical intervention, invasive procedure (intubation, central venous catheter insertion, hemodialysis), and need for vasoactive drugs. 

We had six research assistants review and collect data from these electronic medical records. All six research assistants were trained for data extraction using a standardized data collection form. We tested them for inter-rater reliability using Cohen’s weighted kappa statistics for ordinal scale, and these were more than 0.8 from 10 medical records for bite scene (0.87), type of snake (0.9), bite site (1), skin bleb appearance (1), local reaction (0.88), and management at the ED (0.86), before the data collection. In addition, all 6 assistants were blinded to the objectives of the study; this was decided in an attempt to eliminate selection bias during data collection. 

This study was approved by the Institutional Review Board of the Faculty of Medicine, Vajira Hospital, and the Ethics Committee of the Bangkok Metropolitan Administration (COA 076/2564).

### 6.1. Definitions

Younger adults: Patients aged 15–59 years old.

Older adults: Patients aged ≥60 years old.

Severity of local reaction grading: 1: minimal swelling or no swelling, 2: swelling involves not more than 1 major joint, 3: swelling involves more than 1 major joint but fewer than 2 major joints, 4: swelling involves more than 2 major joints.

GPV bite: A patient was deemed bitten by a GPV when one of the following criteria was met: (1) definite GPV bite: the patient brought in a green pit viper snake for identification or the patient described the snake as having a green body with a red or red-brown tail; (2) probable GPV bite: the patient was bitten by a snake, but could not describe the snakes details, and 2 fang marks were found with signs of local reaction, such as redness or swelling, or there was bleeding from bite marks, and the patient did not have muscle weakness, which is a symptom of neurotoxic snakebites, especially those of cobras, which can be found in Bangkok, and, in addition, if the patient did not see the animal and whether it was a snake but the patient had 2 fang marks with a local reaction and he/she received GPV antivenom because he/she showed signs of systemic effects (abnormal VCT/20WBCT, thrombocytopenia, hypofibrinogenemia, bleeding wounds, or systemic bleeding); (3) possible GPV bite: the patient did not see the animal and whether it was a snake but had 2 fang marks with a local reaction and the patient did not have muscle weakness, which is a symptom of neurotoxic snakes (cobras). In this group, the patient did not receive the GPV antivenom even if he/she had mild coagulopathy/thrombocytopenia. For criterion 2, abnormal bleeding or coagulopathy/thrombocytopenia after an animal bite in Bangkok, especially in our catchment area, is only possible with GPV envenomation [3,28].

Undetermined bite: The patient did not know that he/she was bitten by an animal and only one fang mark or no fang mark was clearly seen. We included this group in this study due to our concern that patients might have been bitten by a GPV without seeing it, as might have happened if it was too dark to identify the snake or if they were bitten in a clump of grass. In addition, we usually treated these patients and had them follow-up as if they were bitten by a GPV because the most concerning envenomation in Bangkok is that from the GPV due to the risk of coagulopathy and systemic bleeding.

Anaphylaxis: Patients had any of these characteristics after bite:

Skin or mucosal symptoms, such as rash, itching, swollen mouth, puffy eyes, red skin, along with respiratory symptoms or hypotension or inadequate blood supply, such as syncope, or urination.

At least 2 of the following symptoms: skin or mucosal manifestations/respiratory symptoms/hypotension or symptoms of poor tissue perfusion/gastrointestinal symptoms.

Hypotension after bite without an alternative explanation, such as coagulopathy with systemic bleeding.

Elapsed time: The length of time the patient was bitten up to the time he/she was triaged in the ED.

Completion for out-patient follow-up: According to the guidelines for management of green pit viper bites published by the Ministry of Public Health of Thailand in 2004, patients are scheduled for a total of 3 follow-up appointments at 24, 48, and 72 h after bite by a GPV. If the patient comes for follow-up all 3 times (at 24 ± 12, 48 ± 12, and 72 ± 12 h), he/she is considered to have had a complete follow-up. We set a time interval of ±12 h because sometimes patients are busy at the time scheduled for the follow-up and are unable to come to their visit on time. Moreover, sometimes when a bite occurs at an unusual time, such as 3 a.m., a follow-up at 3 a.m. seems inappropriate for patients, relatives, and ED providers. Therefore, providing a window of follow-up appointments of ±12 h each day seemed more appropriate.

Clinical outcome: Patient had severe envenomation when he/she had any of the following:

- Abnormal blood test for coagulation: (coagulopathy): VCT > 20 min, or abnormal 20WBCT (unclotted blood at 20 min), platelet count less than 100,000 cells/mm^3^, fibrinogen < 100 mg/dL, or INR >1.2;
- Administration of antivenom: patient received GPV antivenom (F(ab’)2) produced by the Queen Saovabha Memorial Institute (QSMI) if he/she had coagulopathy or abnormal bleeding or signs of impending compartment syndrome/compartment syndrome [25]. The dose was 3–5 vials. The antivenom is a dry powder and needs reconstituting by adding 10 mL of sterile water per vial;
- Administration of blood components: patient received blood components due to abnormal bleeding from the GPV bite;
- If patient had surgical intervention: patient underwent any of the following: incision and drainage, wound debridement, fasciotomy, or limb amputation;
- If patient had an invasive procedure: patient underwent or received any of the following: intubation, central vein catheterization, or hemodialysis;
- If vasopressors were administered: patient received an intravenous infusion of any vasopressor drugs (adrenaline, noradrenaline, dopamine) to improve hemodynamics.

Treatment outcomes included:

- ED revisit: In case of out-patient follow-up, the patient had an ED revisit when he/she returned to the ED without an appointment within 72 ± 12 h;
- Discharge status: In case the patient was admitted to the hospital as an inpatient. The patient’s final status after being treated in the hospital was described as: (1) alive with complete recovery; (2) alive with disability (such as limb loss); (3) alive with permanent neurological deficits, such as muscle weakness (in case of intracranial bleeding); (4) dead (hospital death); or (5) against medical advice.

### 6.2. Statistical Analysis

Quantitative data, such as age, elapsed time from snakebite to ED arrival, pain score, and test results, were reported using means (SDs) or medians (interquartile ranges, IQRs). We compared differences in those continuous data between patients over 60 years old and those under 60 years old using the Student’s *t*-test or the Mann–Whitney U test, with a significance *p*-value of < 0.05.

Qualitative data, such as sex, underlying diseases, geographic location of the bite, bite site body part, bleeding tendency status, and receipt of antivenom, were reported using percentages. We compared the differences in these qualitative data between the elderly (over 60 years old) and the younger (under 60 years old) patients using the chi-squared test or Fisher’s exact test, where appropriate. Data were collected using Microsoft Excel 2019 (Version 2111, Build 16.0.14701.20254). Data were analyzed using IBM SPSS Statistics for Windows, Version 26.0 (IBM Corp, Armonk, NY, USA).

## Figures and Tables

**Table 1:** Patients’ characteristics.

	Total (n= 760)	Ages < 60 (n = 555)	Ages ≥ 60 (n = 205)	*p*-Value
Age (year), median (IQR)	48 (30–61)	40 (26–51)	68 (64–75)	<0.001 *
Male, n (%)	423 (55.7)	322 (58)	101 (49.3)	0.03 *
Underlying, n (%)	218 (28.7)	104 (18.7)	114 (55.6)	<0.001 *
Hypertension	198 (26.1)	92 (16.6)	106 (51.7)	<0.001 *
Diabetes mellitus	94 (12.4)	29 (5.2)	65 (31.7)	<0.001 *
Heart disease	41 (5.4)	11 (2)	30 (14.6)	<0.001 *
Hematologic disease	22 (2.9)	10 (1.8)	12 (5.9)	0.003 *
Liver disease	3 (0.4)	3 (0.5)	0 (0)	0.29
Type of GVP bite, n (%)				0.01 *
GPV bite	721 (94.9)	524 (94.4)	197 (96.0)	
∘ Definite	326 (43.0)	223 (40.2)	103 (50.2)	
∘ Probable	172 (22.6)	140 (25.2)	32 (15.6)	
∘ Possible	223 (29.3)	161 (29.0)	62 (30.2)	
Undetermined	39 (5.1)	31 (5.6)	8 (4.0)	
Current medication, n (%)(only antiplatelets, anticoagulants, or both)	19 (2.5)	5 (0.9)	14 (6.8)	<0.001 *
Bite scene, n (%)				0.33
In door	132 (17.4)	97 (17.5)	35 (17.1)	
Around the house	166 (21.8)	112 (20.2)	54 (26.3)	
Outside away from the house	143 (18.8)	107 (19.3)	36 (17.6)	
No data	319 (42.0)	239 (43.0)	80 (39.0)	
Elapsed time (min), median (IQR)	55 (30–120)	47 (30–118)	69 (35–150)	0.001 *
Elapsed time, n (%)				0.002 *
<30 min	152 (20.0)	122 (22.0)	30 (14.6)	
30–60 min	238 (31.3)	186 (33.5)	52 (25.4)	
60–120 min	187 (24.6)	122 (22.0)	65 (31.7)	
>120 min	183 (24.1)	125 (22.5)	58 (28.3)	
Tetanus vaccination, n (%)				0.56
Completely vaccinated	379 (49.9)	285 (51.3)	94 (45.9)	
Incompletely vaccinated	36 (4.7)	26 (4.7)	10 (4.9)	
Unsure if completely vaccinated	40 (5.3)	26 (4.7)	14 (6.8)	
Never/unknown	111 (14.6)	77 (13.9)	34 (16.5)	
No data	194 (25.5)	141 (25.4)	53 (25.9)	
Vital signs at first ED visit				
Systolic blood pressure, median (IQR)	140 (126–159)	137 (123–152)	154 (135–170)	<0.001 *
Diastolic blood pressure, median (IQR)	84 (75–94)	84 (74–94)	84 (75–93)	0.84
Heart rate, median (IQR)	85 (76–96)	86 (76–97)	80 (74–94)	0.03 *
Respiratory rate, mean (SD)	19 (5)	19 (5)	19 (1)	0.92
Bite site, n (%)				<0.001*
Foot	465 (61.2)	373 (67.2)	92 (44.9)	
Hand	225 (29.6)	131 (23.6)	94 (45.9)	
Other	70 (9.2)	51 (9.2)	19 (9.2)	
Severity of local reactions, n (%)				0.49
Swelling more than 2 major joints	4 (0.5)	2 (0.4)	2 (1.0)	
Involves 2 major joints	31 (4.1)	24 (4.3)	7 (3.4)	
Swelling of no more than 1 major joint	168 (22.1)	118 (21.3)	50 (24.4)	
No swelling or slight swelling	535 (70.4)	397 (71.5)	138 (67.3)	
No data	22 (2.9)	14 (2.5)	8 (3.9)	
Hemorrhagic bleb	20 (2.7)	16 (3.0)	4 (2.0)	0.48
Wound necrosis	6 (0.8)	5 (0.9)	1 (0.5)	0.57
Other systemic symptoms from envenomation (before receipt of treatment)				
Rash	1 (0.1)	1 (0.2)	0 (0.0)	0.54
Dyspnea, chest pain	2 (0.2)	1 (0.2)	1 (0.5)	0.46
Nausea, vomiting	5 (0.7)	5 (0.9)	0 (0.0)	0.33
Non-guarding abdominal pain	2 (0.2)	1 (0.2)	1 (0.5)	0.46

* Significant at *p*-value < 0.05.

**Table 3:** Compliance of out-patient follow-ups in GPV bite cases.

Follow-Up Compliance	Total ^†^ n= 674	Ages < 60 n = 491	Ages ≥ 60 n=183	*p*-Value
Complete	238 (35.3)	159 (32.4)	79 (43.2)	0.01 *
Incomplete	436 (64.7)	332 (67.6)	104 (56.8)	

^†^ Excluded admitted cases/referral cases/cases left against medical advice. * Significant at *p-value* < 0.05

**Table 4:** Details of incomplete follow-up.

Details of Incomplete Follow-Up	Total ^†^ (n = 436)	Ages < 60 (n = 332)	Ages ≥ 60 (n = 104)	*p*-Value
Loss to follow-up at 24 ± 12 h	116 (26.6)	94 (28.3)	22 (21.2)	0.04 *
Loss to follow-up at 48 ± 12 h	190 (43.6)	149 (44.9)	41 (39.4)	
Loss to follow-up at 72 ± 12 h	130 (29.8)	89 (26.8)	41 (39.4)	

^†^ Excluded admitted cases/referral cases/cases left against medical advice. * Significant at *p-value* < 0.05

## Data Availability

Not applicable.

## Supplementary Materials

The following supporting information can be downloaded at: https://www.mdpi.com/article/10.3390/toxins14120869/s1, Table S1: Age distribution of the population in this study; Table S2: Abnormal laboratory results; Table S3: Compliance of follow-up sorted by hospital; Table S4: Compliance between 2 hospitals; Table S5: Select Patients’ characteristics stratified by certainty of GPV identification: Table S6: Clinical and treatment outcomes stratified by certainty of GPV identification; Table S7: Compliance of outpatient follow-up in GPV bite cases stratified by certainty of GPV identification.
