# Supplementary material for: Green Pit Viper Envenomations in Bangkok: A Comparison of Follow-Up Compliance and Clinical Outcomes in Older and Younger Adults
Source: Toxins (Basel). 2022 Dec 10;14(12):869. doi: 10.3390/toxins14120869 (PMC9784995; doi:10.3390/toxins14120869)
Supplement: Supplementary file 1 [file toxins-14-00869-s001.zip › toxins-1978923-Supplementary.pdf]

Green Pit Viper Envenomations in Bangkok: A Comparison of Follow-up Compliance and Clinical Outcomes in Older and Younger Adults

Rittirak Othong, Thanaphat Eurcherdkul and Prasit Chantawatsharakorn

Table S1. Age distribution of the population in this study.

Age group	Total
15-19	65
20-29	133
30-39	93
40-49	124
50-59	140
60-69	123
70-79	61
80-86	21
Total	760

Table S2: Abnormal laboratory results.

	**Total**		**Undetermined**		**Suspected GPV**	
	**Ages** < 60 **years** (**n**=555)	**Ages**≥ 60 **years** (**n**=205)	**Ages** < 60 **years** (**n**=31)	**Ages**≥ 60 **years** (**n**=8)	**Ages** < 60 **years** (**n**=524)	**Ages**≥ 60 **years** (**n**=197)
PLT < 100,000						
• 1st time	10 (1.8)	6 (2.9)	0 (0)	0 (0)	10 (1.9)	6 (3.1)
• 2nd time	6 (1.1)	3 (1.5)	0 (0)	0 (0)	6 (1.2)	3 (1.5)
• 24±12 h.	8 (1.4)	2 (1)	1 (3.2)	0 (0)	7 (1.3)	2 (1)
• 48±12 h.	3 (0.5)	1 (0.5)	0 (0)	0 (0)	3 (0.6)	1 (0.5)
• 72±12 h.	3 (0.5)	1 (0.5)	0 (0)	0 (0)	3 (0.6)	1 (0.5)
VCT > 20						
• 1st time	16 (2.9)	10 (4.9)	2 (6.5)	0 (0)	14 (2.7)	10 (5.1)
• 2nd time	14 (2.5)	5 (2.4)	0 (0)	0 (0)	14 (2.7)	5 (2.5)
• 24±12 h.	14 (2.5)	0 (0)	0 (0)	0 (0)	14 (2.7)	0 (0)
• 48±12 h.	3 (0.5)	1 (0.5)	0 (0)	0 (0)	3 (0.6)	1 (0.5)
• 72±12 h.	0 (0)	2 (1)	0 (0)	0 (0)	0 (0)	2 (1)
Fibrinogen level < 100						
• 1st time	4 (0.7)	1 (0.5)	0 (0)	0 (0)	4 (0.8)	1 (0.5)
• 2nd time	1 (0.2)	0 (0)	0 (0)	0 (0)	1 (0.2)	0 (0)
• 24±12 h.	0 (0)	0 (0)	0 (0)	0 (0)	0 (0)	0 (0)
• 48±12 h.	0 (0)	0 (0)	0 (0)	0 (0)	0 (0)	0 (0)
• 72±12 h.	0 (0)	0 (0)	0 (0)	0 (0)	0 (0)	0 (0)
INR > 1.2						
• 1st time	11 (2)	8 (3.9)	1 (3.2)	0 (0)	10 (1.9)	8 (4.1)
• 2nd time	2 (0.4)	1 (0.5)	0 (0)	0 (0)	2 (0.4)	1 (0.5)
• 24±12 h.	1 (0.2)	0 (0)	0 (0)	0 (0)	1 (0.2)	0 (0)
• 48±12 h.	3 (0.5)	0 (0)	0 (0)	0 (0)	3 (0.6)	0 (0)

•	72±12 h.	1 (0.2)	3 (1.5)	0 (0)	0 (0)	1 (0.2)	3 (1.5)

Table S3: Compliance of follow-up sorted by hospital†.

**Follow-up compliance, including undetermined group**	**Vajira hospital (n=440)**	**Taksin hospital (n=234)**	**P-value**
• Complete	213 (48.4)	25 (10.7)	<0.001*
• Incomplete	227 (51.6)	209 (89.3)	
Follow-up compliance, excluding undetermined group	Vajira hospital (n=414)	Taksin hospital (n=224)	P-value
• Complete	205 (49.5)	24 (10.7)	<0.001*
• Incomplete	209 (50.5)	200 (89.3)	

†Excluded admitted cases/referral cases/cases left against medical advice, *Significant at P-value < 0.05

Table S4: Compliance between 2 hospitals.

	**Vajira hospital**	**Taksin hospital**	**P-value**
Compliance of follow-up			
• Complete	213 (48.4)	25 (10.7)	<0.001*
• Incomplete	227 (51.6)	209 (89.3)	
- Loss to follow-up at 24±12 h.	92 (40.5)	24 (11.6)	<0.001*
- Loss to follow-up at 48±12 h.	78 (34.4)	112 (53.6)	
- Loss to follow-up at 72±12 h.	57 (25.1)	73 (34.8)	

*Significant at P-value < 0.05

Table S5: Select Patients' characteristics stratified by certainty of GPV identification.

Table S5.1: Definite GPV bite.

	**Total (n=326)**	**Ages <** 60 **(n=223)**	**Ages ≥** 60 **(n=102)**	**P-value**
Elapsed time since bite (min), median (IQR)	49.5 (30 - 109)	45 (30 - 90)	80 (35 - 159)	<0.001*
Elapsed time, n (%)				0.002*
• < 30 min	72 (22.1)	54 (24.2)	18 (17.5)	
• 30-60 min	105 (32.2)	81 (36.3)	24 (23.3)	
• 60-120 min	84 (25.8)	55 (24.7)	29 (28.2)	
• > 120 min	65 (19.9)	33 (14.8)	32 (31.1)	
Severity of local reactions, n (%)				0.80
• Swelling more than 2 major joint	3 (0.9)	2 (0.9)	1 (1)	
• Involves 2 major joint	13 (4)	9 (4)	4 (3.9)	
• Swelling of no more than 1 major joint	90 (27.6)	58 (26)	32 (31.1)	
• Not swelling or slightly	216 (66.3)	152 (68.2)	64 (62.1)	
• No data	4 (1.2)	2 (0.9)	2 (1.9)	
Hemorrhagic bleb	14 (4.3)	12 (5.4)	2 (1.9)	0.24
Wound necrosis	5 (1.5)	4 (1.8)	1 (1)	0.57

*Significant at P-value < 0.05

Table S5.2: Probable GPV bite.

	Total (n=172)	Ages < 60 (n=140)	Ages ≥ 60 (n=32)	P-value
Elapsed time since bite (min), median (IQR)	57.5 (30 - 120)	45 (30 - 112.5)	84.5 (38.5 - 143.5)	0.03*
Elapsed time, n (%)				0.045*
• < 30 min	33 (19.2)	30 (21.4)	3 (9.4)	
• 30-60 min	53 (30.8)	46 (32.9)	7 (21.9)	
• 60-120 min	44 (25.6)	30 (21.4)	14 (43.8)	
• > 120 min	42 (24.4)	34 (24.3)	8 (25)	
Severity of local reactions, n (%)				0.13
• Swelling more than 2 major joint	1 (0.6)	0 (0)	1 (3.1)	
• Involves 2 major joint	16 (9.3)	13 (9.3)	3 (9.4)	
• Swelling of no more than 1 major joint	31 (18)	28 (20)	3 (9.4)	
• Not swelling or slightly	121 (70.4)	96 (68.6)	25 (78.1)	
• No data	3 (1.7)	3 (2.1)	0 (0)	
Hemorrhagic bleb	3 (1.7)	2 (1.4)	1 (3.1)	0.46
Wound necrosis	0	0	0	

*Significant at P-value < 0.05

Table S5.3: Possible GPV bite.

	Total (n=223)	Age < 60 (n=161)	Age ≥ 60 (n=62)	P-value
Elapsed time since bite (min), median (IQR)	52 (30 - 125)	50 (30 - 122)	61 (30 - 150)	0.71
Elapsed time, n (%)				0.74
• < 30 min	42 (18.8)	33 (20.5)	9 (14.5)	
• 30-60 min	72 (32.3)	52 (32.3)	20 (32.3)	
• 60-120 min	51 (22.9)	35 (21.7)	16 (25.8)	
• > 120 min	58 (26)	41 (25.5)	17 (27.4)	
Severity of local reactions, n (%)				0.40
• Swelling more than 2 major joint	0	0	0	
• Involves 2 major joint	2 (0.9)	2 (1.2)	0 (0)	
• Swelling of no more than 1 major joint	41 (18.4)	28 (17.4)	13 (21)	
• Not swelling or slightly	166 (74.4)	123 (76.4)	43 (69.4)	
• No data	14 (6.3)	8 (5)	6 (9.7)	
Hemorrhagic bleb	3 (1.4)	2 (1.2)	1 (1.6)	0.83
Wound necrosis	0	0	0	NA

Table S5.4: Undetermined GPV bite.

	Total (n=39)	Ages < 60 (n=31)	Ages ≥ 60 (n=8)	P-value
Elapsed time since bite (min), median (IQR)	85 (42 - 249)	130 (30 - 257)	68 (60 - 91)	0.66
Elapsed time, n (%)				0.001*
• < 30 min	5 (12.8)	5 (16.1)	0 (0)	
• 30-60 min	8 (20.5)	7 (22.6)	1 (12.5)	
• 60-120 min	8 (20.5)	2 (6.5)	6 (75)	
• > 120 min	18 (46.2)	17 (54.8)	1 (12.5)	
Severity of local reactions, n (%)				0.63
• Swelling more than 2 major joint	0	0	0	
• Involves 2 major joint	0	0	0	
• Swelling of no more than 1 major joint	6 (15.4)	4 (12.9)	2 (25)	

• Not swelling or slightly	32 (82.1)	26 (83.9)	6 (75)	
• No data	1 (2.6)	1 (3.2)	0 (0)	
Hemorrhagic bleb	0	0	0	NA
Wound necrosis	1 (2.6)	1 (3.2)	0 (0)	0.61

*Significant at P-value < 0.05

Table S6: Clinical and treatment outcomes stratified by certainty of GPV identification.

Supplementary Table S6.1: Definite GPV bite

**Clinical outcome**	**Total** (**n=326**)	**Age** < 60 (**n=223**)	**Age** ≥ 60 (**n=103**)	**P-value**
Abnormal labs (at any time, any lab)	39 (12)	28 (12.6)	11 (10.7)	0.63
- Abnormal VCT or 20WBCT	24 (7.4)	17 (7.6)	7 (6.8)	0.79
- Abnormal platelet (<100,000)	6/305 (2)	3/208 (1.4)	3/97 (3.1)	0.33
- Abnormal fibrinogen(<100)	2/35 (5.7)	2/26 (7.7)	0/9 (0)	0.39
- Abnormal INR (>1.2)	11/209 (5.3)	9/146 (6.2)	2/63 (3.2)	0.51
Received antivenom	21 (6.4)	12 (5.4)	9 (8.7)	0.25
Treatment outcome				
Disposition after initial ED management				
• Observation (details of disposition after ED observation are below)	318 (97.6)	217 (97.3)	101 (98.1)	0.89
• Discharge	2 (0.6)	1 (0.5)	1 (1)	
• Hospital admission	5 (1.5)	4 (1.8)	1 (1)	
• Referral	1 (0.3)	1 (0.5)	0 (0)	
Disposition after ED observation	n=318	n=217	n=101	
• Discharge	295 (92.8)	206 (94.9)	89 (88.1)	0.09
• Hospital admission	11 (3.5)	5 (2.3)	6 (5.9)	
• Referral	12 (3.8)	6 (2.8)	6 (5.9)	

Table S6.2: Probable GPV bite.

**Clinical outcome**	**Total** (**n=172**)	**Age** < 60 (**n=140**)	**Age** ≥ 60 (**n=32**)	**P-value**
Abnormal labs (at any time, any lab)	17 (9.9)	15 (10.7)	2 (6.3)	0.44
- Abnormal VCT or 20WBCT	12 (7)	12 (8.6)	0 (0)	0.13
- Abnormal platelet (<100,000)	4/157 (2.6)	3/129 (2.3)	1/28 (3.6)	0.55
- Abnormal fibrinogen(<100)	0	0	0	
- Abnormal INR (>1.2)	1/113 (0.9)	0/93 (0)	1/20 (5)	0.18
Received antivenom	17 (9.9)	15 (10.7)	2 (6.3)	0.44
Treatment outcome				
Disposition after initial ED management				
• Observation (details of disposition after ED observation are below)	165 (95.9)	133 (95)	32 (100)	0.67
• Discharge	1 (0.6)	1 (0.7)	0 (0)	
• Hospital admission	6 (3.5)	6 (4.3)	0 (0)	
Disposition after ED observation	N=165	N=133	N=32	
• Discharge	147 (89.1)	117 (88)	30 (93.8)	0.49
• Hospital admission	10 (6.1)	8 (6)	2 (6.3)	

• Referral	8 (4.9)	8 (6)	0 (0)	

Table S6.3: Possible GPV bite.

Clinical outcome	**Total (n=223)**	**Age <** 60 (**n=161**)	**Age** ≥ 60 (**n=62**)	**P-value**
Abnormal labs (at any time, any lab)	21 (9.4)	17 (10.6)	4 (6.5)	0.45
- Abnormal VCT or 20WBCT	15 (6.7)	14 (8.7)	1 (1.6)	0.07
- Abnormal platelet (<100,000)	4/210 (1.9)	1/151 (0.7)	3/59 (5.1)	0.07
- Abnormal fibrinogen(<100)	2/23 (8.7)	2/16 (12.5)	0/7 (0)	0.33
- Abnormal INR (>1.2)	5/153 (3.3)	5/112 (4.5)	0/41 (0)	0.33
Received antivenom	0	0	0	NA
Treatment outcome				
Disposition after initial ED management				
• Observation (details of disposition after ED observation are below)	217 (97.3)	155 (96.3)	62 (100)	0.84
• Discharge	3 (1.4)	3 (1.9)	0 (0)	
• Hospital admission	1 (0.5)	1 (0.6)	0 (0)	
• Referral	1 (0.5)	1 (0.6)	0 (0)	
• Left against medical advise	1 (0.5)	1 (0.6)	0 (0)	
Disposition after ED observation	n=217	n=155	n=62	
• Discharge	190 (87.6)	135 (87.1)	55 (88.7)	0.94
• Hospital admission	7 (3.2)	5 (3.2)	2 (3.2)	
• Referral	20 (9.2)	15 (9.7)	5 (8.1)	

Table S6.4: Undetermined GPV bite.

Clinical outcome	**Total (n=39)**	**Age <** 60 (**n=31**)	**Age** ≥ 60 (**n=8**)	**P-value**
Abnormal labs (at any time, any lab)	6 (15.4)	5 (16.1)	1 (12.5)	0.80
- Abnormal VCT or 20WBCT	3 (7.7)	3 (9.7)	0 (0)	0.36
- Abnormal platelet (<100,000)	2/35 (5.7)	2/28 (7.1)	0/7 (0)	0.47
- Abnormal fibrinogen(<100)	1/3 (33.3)	0/1 (0)	1/2 (50)	0.39
- Abnormal INR (>1.2)	2/27 (7.4)	2/20 (10)	0/7 (0)	0.39
Received antivenom	1 (2.6)	1 (3.2)	0 (0)	0.61
Treatment outcome				
Disposition after initial ED management				
• Observation (details of disposition after ED observation are below)	38 (97.4)	30 (96.8)	8 (100)	0.61
• Hospital admission	1 (2.6)	1 (3.2)	0 (0)	
Disposition after ED observation	N=38	N=30	N=8	
• Discharge	36 (94.7)	28 (93.3)	8 (100)	0.45
• Hospital admission	2 (5.3)	2 (6.7)	0 (0)	

Table S7: Compliance of outpatient follow-up in GPV bite cases stratified by certainty of GPV identification.

**Follow-up compliance**	**Total**[†]	**Ages** < 60	**Ages** ≥ 60	**P-value**
Definite	n=285	n=196	n=89	0.42
• Complete	96 (33.7)	69 (35.2)	27 (30.3)	
• Incomplete	189 (66.3)	127 (64.8)	62 (69.7)	
Probable	n=155	n=127	n=28	0.43
• Complete	60 (38.7)	51 (40.2)	9 (32.1)	
• Incomplete	95 (61.3)	76 (59.8)	19 (67.9)	
Possible	n=201	n=143	n=58	0.98
• Complete	70 (34.8)	50 (35)	20 (34.5)	
• Incomplete	131 (65.2)	93 (65)	38 (65.5)	
Undetermined	n=33	n=27	n=6	0.87
• Complete	12 (36.4)	10 (37)	2 (33.3)	
• Incomplete	21 (63.6)	17 (63)	4 (66.7)	
